# Caffeine Citrate for Apnea of Prematurity: A Prospective, Open-Label, Single-Arm Study in Chinese Neonates

**DOI:** 10.3389/fped.2020.00076

**Published:** 2020-03-11

**Authors:** Lizhong Du, Xiaomei Tong, Chao Chen, Xirong Gao, Alessandra Gagnatelli, Jingyang Li, Debora Santoro, Sara Nicolardi, Laura Fabbri, The Peyona Chinese Study Group

**Affiliations:** ^1^Neonatal Intensive Care Unit, Children's Hospital of Zhejiang University School of Medicine, Hangzhou, China; ^2^Peking University Third Hospital, Beijing, China; ^3^Fudan University Children's Hospital, Shanghai, China; ^4^Hunan Children's Hospital, Changsha, China; ^5^Research & Development – Neonatology, Chiesi Farmaceutici SpA, Parma, Italy; ^6^Chiesi Pharmaceutical (Shanghai) Co., Ltd., Shanghai, China

**Keywords:** infant, newborn, drug therapy, bodyweight, apnea

## Abstract

**Background:** Caffeine citrate has been approved in China for the management of apnea of prematurity. This clinical trial was conducted as a condition of regulatory approval. The aim was to confirm the efficacy of caffeine citrate in the treatment of recurrent intermittent hypoxia and bradycardia in preterm newborns with primary apnea.

**Objectives:** The primary outcome was the change from baseline in the number of apnea events after loading dose administration of caffeine citrate. Secondary efficacy outcomes included the change from baseline in apnea events after 2 and 4 weeks of maintenance doses.

**Methods:** This was a multicenter, prospective longitudinal open-label, single-arm study. Neonates who had experienced at least four apnea events during a 24 h period received a loading dose of caffeine citrate 20 mg/kg; those who required additional maintenance doses received 5 mg/kg/day (titrated up to 10 mg/kg/day in case of insufficient response). The number of apnea events was recorded for 6–12 h prior to the loading dose (baseline), and for 12 h post-dose, following the loading dose and at Weeks 2 and 4 (during maintenance).

**Results:** A total of 247 neonates received the loading dose, who had a significant reduction from baseline of 3.9 events (p < 0.001) in the mean number of apnea events. The subset of neonates who required maintenance doses also had significant reductions in the number of events at all visits (p < 0.001 for all). A total of 79.4% of participants had at least one adverse event, but only one non-serious and no serious events were considered related to treatment.

**Conclusions:** In this large, prospective, open-label study, premature infants with a history of apnea who received caffeine citrate were significantly less likely to experience further apnea events.

## Introduction

The incidence of apnea of prematurity increases as gestational age decreases, from 7% of neonates born at 34–35 weeks to nearly 100% of those born before 29 weeks (1). This contributes substantially to the length of hospitalization (2). Severe apnea (lasting longer than 20 s) is usually associated with bradycardia or desaturation, which may in turn lead to disturbances of cerebral hemodynamics, subsequently impacting neurodevelopment (1). Furthermore, in a *post-hoc* analysis of data from extremely preterm neonates, prolonged hypoxemic episodes during the first 3 months after birth were associated with a range of adverse outcomes, including increased mortality after 36 weeks, motor impairment, cognitive or language delay, severe hearing loss, and bilateral blindness (3).

Methylxanthine therapy is the mainstay of pharmacologic therapy for apnea of prematurity (4, 5). Two forms are predominantly used, caffeine citrate and theophylline, both of which have similar efficacy, although caffeine citrate is associated with a better safety profile and a lower incidence of adverse events (6, 7). Further, compared with theophylline, caffeine citrate has a longer half-life and does not require drug-level monitoring, and is therefore described in guidelines as generally preferred (4). However, most of the data supporting these treatment guidelines are from relatively small, old studies (8–11), with only one large, long-term follow-up study (12, 13).

Although caffeine citrate has been used for the management of apnea of prematurity in Europe and the US for decades, it was approved in China in December 2012. As a condition of the regulatory approval, the manufacturer was asked to conduct a clinical trial to evaluate the efficacy and possible side-effects of caffeine citrate in neonates who were experiencing apnea and who were being managed under current best clinical practice. The aim was to confirm the efficacy of caffeine citrate in the treatment of recurrent intermittent hypoxia and bradycardia in preterm newborns with primary apnea.

## Methods

### Trial Design

**Figure 1:**
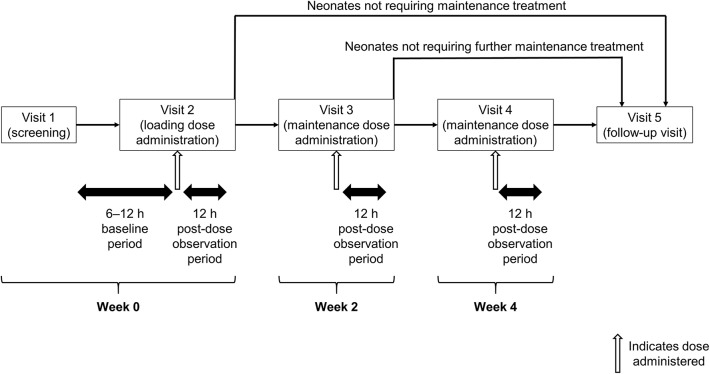
Study design.

This was a multicenter, prospective longitudinal open-label, single-arm study that included five visits (Figure 1). At a screening visit (Visit 1), after parents or legal guardians provided written informed consent, neonates who met the inclusion/exclusion criteria had their demographic and medical history collected, and their baseline bodyweight recorded. Baseline apnea data were collected between Visits 1 and 2, including related pulse-oximetry for transcutaneous oxygen saturation (SpO_2_) and cardiopulmonary monitoring using chest electrodes to record heart rate and respiratory rate/apnea. At Visit 2, neonates received a loading dose of caffeine citrate and were then observed for a further 12 h. Between Visits 2 and 4, neonates received maintenance caffeine citrate, continuing until they reached an age of 37 weeks or had 5–7 days without significant apnea events (where significant apnea events were those accompanied by desaturation <80% SpO_2_ and/or bradycardia <100 bpm). Visits 3 and 4 took place 2 and 4 weeks after Visit 2, respectively, and commenced 12 h prior to dosing with caffeine citrate, running until 12 h post-dose. Visit 5 was a follow-up visit, taking place 5 days after discontinuation of caffeine citrate therapy.

The study was approved by the independent ethics committee at each institution (see Supplementary Material), and was performed in accordance with the principles of the Declaration of Helsinki, and the International Conference on Harmonization notes for guidance on Good Clinical Practice (ICH/CPMP/135/95). The protocol was amended twice after commencement of recruitment, mainly to match the inclusion/exclusion criteria to standard clinical practice in the study sites, to reduce the number of apnea events required for inclusion. The only criterion added was to exclude neonates with any condition that, in the opinion of the investigator, made them unsuitable for participation in the study. In response to requests from ethics committees and study sites the baseline observation period was shortened to commence a minimum of 6 h prior to loading dose administration, rather than 12 h pre-dose. The study is registered at www.chinadrugtrials.org.cn (CTR20140706).

### Participants

Eligible patients were male or female, gestational age 28–33 weeks, with a first apnea event resulting in breathing cessation for ≥20 s, or accompanied by bradycardia (heart rate <100 bpm) or oxygen desaturation (SpO_2_ <80%), and that occurred more than 12 h after birth. Furthermore, eligible neonates had at least three other apnea events within 24 h after the first occurrence. The main reasons for exclusion were: hematocrit >65% or <40% in room air; clinical suspicion or proven sepsis; blood urea nitrogen >20 mg/dL or urine output <1 mL/kg/h; body temperature <36.0 or >38.5C; hemodynamically significant patent ductus arteriosus confirmed by cardiac ultrasound; suspected or confirmed necrotizing enterocolitis; or confirmed intraventricular hemorrhage above Grade 2. Full inclusion and exclusion criteria are listed in the Supplementary Material.

### Intervention

Caffeine citrate (Peyona^®^, Chiesi Farmaceutici SpA) was administered at Visit 2 in a loading dose of 20 mg/kg bodyweight via controlled intravenous infusion over 30 min, using a syringe infusion pump. The maintenance dose was 5 mg/kg bodyweight every 24 h, orally or by intravenous infusion. This maintenance dose could be titrated by the investigator up to 10 mg/kg/day if the neonate responded poorly. The protocol did not mandate whether continuous positive airway pressure or non-invasive positive-pressure ventilation should be used and did not prevent switching from one to the other.

### Outcomes

The primary objective was to confirm the efficacy of caffeine citrate in the treatment of recurrent intermittent hypoxia and bradycardia in preterm newborns with primary apnea. The primary efficacy outcome was the change from baseline in the number of apnea events, accompanied by desaturation <80% SpO_2_ and/or bradycardia <100 bpm, during the 12 h after the loading dose administration at Visit 2, as documented during continuous electronic pulse-oximeter recording. The baseline value was the number of apnea events that occurred during the 6–12 h between Visit 1 and administration of the loading dose at Visit 2.

Secondary efficacy outcomes included the change from baseline in apnea events at Visits 3 and 4, the proportion of neonates with ≥50% reduction from baseline in apnea events at each visit, and the change from baseline in bodyweight at Visits 3 and 4. Adverse events were recorded throughout the study, including at the follow-up visit (Visit 5).

### Sample Size

This study was not formally powered, due to the lack of reference data. Regulatory requirements (as part of the approval of caffeine citrate in China) were for a study to be conducted with not less than 200 neonates. To cover a potential drop-out of 20%, the final planned sample size was 240 neonates, from approximately 20 neonatal intensive care units.

### Statistical Methods

The number of apnea events at Visits 2, 3 and 4 were compared to baseline using the Wilcoxon signed rank-test. As the period over which the baseline data were collected (Visits 1 to 2) could vary, the baseline value was weighted based on actual monitoring time, and was then standardized to 12 h. Bodyweight at each visit was compared to baseline by means of a paired *t*-test. The percentage of neonates with ≥50% reduction from baseline in the number of apnea events was summarized descriptively.

The safety set included all neonates who received at least one administration of caffeine citrate. The full analysis set included all neonates in the safety set who had at least one available post-baseline efficacy evaluation, and the per protocol set included all neonates from the full analysis set without any major protocol deviations. The primary objective was analyzed both in the full analysis set and the per protocol set. The other efficacy variables were analyzed in the full analysis set only, with the safety data analyzed in the safety set.

## Results

### Participants

The study was conducted between June 2014 and October 2015 in 19 neonatal intensive care units, all in China. Of 259 neonates screened, 248 were enrolled, with 247 receiving the caffeine citrate loading dose; these neonates comprised both the safety analysis set and the full analysis set, the baseline characteristics of whom are reported in Table 1. A total of 203 (81.9%) participants completed Visit 2. The reasons for withdrawal of the 45 neonates were adverse events (*n* = 15), treatment failure (*n* = 14), consent withdrawal (*n* = 5), hematocrit out of range (*n* = 3), abnormal test results (*n* = 2), requirement for assisted ventilation via an endotracheal tube or intermittent mandatory ventilation (*n* = 1) and “other” (*n* = 5). The Visit 3 (Week 2) analyses included 80 neonates, with 26 neonates included in the Visit 4 (Week 4) analyses.

**Table 1:** Baseline characteristics (safety analysis set).

	**Neonates (*N* = 247)**
**Gender, n (%)**	
Male	147 (59.5%)
Female	100 (40.5%)
**Race, n (%)**	
Han nationality	238 (96.4%)
Other	9 (3.6%)
**Gestational age, weeks**	
Mean (SD)	30.9 (1.45)
Min, max	28, 35
**Weight, g**	
Mean (SD)	1489.1 (317.88)
Min, max	680, 2,200

### Outcomes

For the primary endpoint, in the full analysis set, there was a significant reduction in the mean number of apnea events from baseline to Visit 2 of 3.9 events (Table 2). The results for the per protocol set were consistent with those for the full analysis set, with a mean change from baseline of −4.0 (range −25.3 to 4.6; *P* < 0.001). The subset of neonates who required maintenance doses also had significant reductions in the number of events at all visits (*P* < 0.001 for all). For example, the 80 neonates treated at Visit 3 had mean reductions from baseline of 3.9 events at Visit 2 and 4.8 events at Visit 3 (Table 2).

**Table 2:** Events of apnea per 12 h throughout the study, together with changes from baseline (full analysis set).

	**Population included in Visit 2 analyses (*N* = 247)**	**Population included in Visit 3/Week 2 analyses (*N* = 80)**	**Population included in Visit 4/Week 4 analyses (*N* = 26)**
Baseline^*^			
Mean (SD)	5.0 (3.49)	5.2 (2.96)	5.1 (2.78)
Range	1.0–25.3	1.3–19.2	2–10.9
**Visit 2**			
Mean (SD)	1.1 (1.76)	1.3 (2.04)	1.5 (1.70)
Range	0–12	0–12	0–6
**Change from baseline at Visit 2**			
Mean (SD)	−3.9 (3.73)	−3.9 (3.34)	−3.5 (2.14)
Range	−25.3 to 4.6	−19.2 to 3.96	−8 to 1
95% CI	−4.4 to −3.5	−4.6 to −3.1	−4.4 to −2.6
*P*-value	<0.001	<0.001	<0.001
**Visit 3**			
Mean (SD)		0.4 (1.16)	0.7 (1.85)
Range		0, 9	0, 9
**Change from baseline at Visit 3**			
Mean (SD)		−4.8 (3.22)	−4.3 (3.51)
Range		−19.2 to 4.0	−11.0 to 4.0
95% CI		−5.6 to −4.1	−5.7 to −2.9
*P*-value		<0.001	<0.001
**Visit 4**			
Mean (SD)			0.4 (1.03)
Range			0, 4
**Change from baseline at Visit 4**			
Mean (SD)			−4.6 (2.66)
Range			−10.5 to −0.5
95% CI			−5.7 to −3.5
*P*-value			<0.001

* *Baseline values are standardized to a 12 h period*.

Most neonates at all visits had at least a 50% reduction in the number of apnea events (Visit 2, 200/247 [81.0%]; Visit 3, 77/80 [96.3%]; Visit 4, 24/26 [92.3%]). Furthermore, for the subgroup requiring maintenance therapy, there was a significant overall increase in bodyweight at both Visits 3 and 4 (*P* < 0.001 for all; Table 3).

**Table 3:** Bodyweight, together with changes from baseline (full analysis set).

**Bodyweight, g**	**Population included in Visit 3/Week 2 analyses (*N* = 80)**	**Population included in Visit 4/Week 4 analyses (*N* = 26)**
**Baseline**		
Mean (SD)	1,299 (245.3)	1,248 (239.7)
Range	700–2,000	770–1,690
**Visit 3**		
Mean (SD)	1,493 (275.0)	1,430 (241.5)
Range	810–2,150	960–1,890
**Change from baseline at Visit 3**		
Mean (SD)	194 (128.5)	183 (129.4)
Range	−210 to 570	−210 to 440
95% CI	166–223	130–235
*P*-value	<0.001	<0.001
**Visit 4**		
Mean (SD)		1,748 (262.1)
Range		1,160–2,320
**Change from baseline at Visit 4**		
Mean (SD)		500 (162.9)
Range		120–920
95% CI		435–566
*P*-value		<0.001

### Safety

Only one non-serious event was considered related to treatment (sinus tachycardia, which resolved spontaneously without treatment after study drug was discontinued), with no serious adverse events considered related to treatment (Table 4). Although 9 (3.6%) neonates died during the study, no deaths were considered related to caffeine citrate therapy.

**Table 4:** Overall experience of adverse events, including adverse events occurring in >5% of neonates, important adverse events occurring in >2% of neonates, and serious adverse events occurring in >0.5% of neonates (safety set).

***n* (%)**	**Neonates (*N* = 247)**
**Any adverse event**	196 (79.4)
Anemia	82 (33.2)
Sepsis	26 (10.5)
Pneumonia	22 (8.9)
Infection	14 (5.7)
Necrotizing enterocolitis	14 (5.7)
Hypocalcemia	17 (6.9)
Jaundice	41 (16.6)
Atrial septal defect	20 (8.1)
Patent ductus arteriosus	19 (7.7)
**Any treatment-related adverse event**	1 (0.4)
**Any serious adverse event**	34 (13.8)
Sepsis	15 (6.1)
Necrotizing enterocolitis	14 (5.7)
Apnea	2 (0.8)
**Adverse event leading to discontinuation**	21 (8.5)
**Adverse event leading to death**	9 (3.6)

## Discussion

This is largest caffeine citrate trial in Chinese preterm neonates to date. The trial documented the efficacy and safety of caffeine citrate for apnea of prematurity. An initial loading dose of caffeine citrate significantly reduced the number of subsequent apnea events in a group of neonates who had experienced at least four apnea events since birth, with almost all of the participants having at least a 50% reduction in the number of events, confirming the rapid onset of the efficacy of caffeine citrate. Those neonates who received maintenance therapy had an overall reduction in apnea events at all visits and had significant improvements in bodyweight. Furthermore, caffeine citrate had a good overall safety profile, since although more than three-quarters of neonates had at least one adverse event, the majority were as expected for this population, with only one event considered related to treatment.

The results of our study are broadly consistent with previous studies conducted outside China. In one of these, 18 neonates were randomly assigned to a treatment or control group for 15 days (9). The nine neonates who received caffeine citrate had a significant decrease from baseline in apnea, with this benefit observed from the first day of treatment; those in the control group had no improvement in apnea for the duration of the study. In a second study, 85 neonates were randomized to receive caffeine citrate or a placebo for up to 10 days, with caffeine citrate again associated with a rapid improvement in apnea and the difference vs. the placebo treatment approaching significance within 2 days (10).

These relatively small early studies were then followed by the Caffeine for Apnea of Prematurity (CAP) study, in which over 2,000 neonates were randomized to receive either caffeine citrate or a placebo (12). During the first 3 weeks after randomization, neonates receiving caffeine citrate gained less weight than those in the placebo group, with a mean decrease from the baseline. Indeed, failure to thrive and feeding intolerance are recognized as adverse reactions of caffeine citrate (although of unknown incidence) (14), with one study suggesting that long-term administration of caffeine in preterm neonates being associated with an increase in oxygen consumption and a consequent reduction in weight gain (15). The bodyweight data from the current study are therefore especially reassuring, given that the majority of the neonates gained weight between the baseline and Visit 3 (Week 2), with all neonates gaining weight between the baseline and Visit 4.

The primary endpoint of the current study, change from baseline in the number of apnea events following the initial loading dose, was assessed in the overall population, all of whom had experienced at least four apnea events over a 24 h period. Such infants are at risk of a range of long-term negative consequences including neurological development (1). One of the few studies examining the long-term benefits of caffeine citrate in neonates is the CAP trial, in which patients were followed up with at the age of 11 years (12). Those who received caffeine citrate as a neonate had an improved expiratory flow (16) and a reduced risk of motor impairment (13) at follow-up compared with those neonates who did not receive caffeine.

The overall safety profile of caffeine citrate seen in this study is consistent with that in a large post-authorization safety study, which evaluated the clinical use, outcomes, and the safety profile in 506 neonates (17). Adverse drug reactions were reported in 4.2% of the neonates; the only event to occur in more than 1% of neonates was tachycardia (in 2.4%), none of which was considered serious.

Given all the infants in this study were being managed according to current best (international) clinical practices (and indeed the study protocol was amended to ensure it met these conditions), the results should be generalizable. However, an obvious limitation of this study is the lack of a control arm, especially in terms of the interpretation of some of the secondary endpoints. The use of a placebo comparator would have been unethical in this population, given caffeine citrate is approved and is standard in the care of apnea in neonates. Furthermore, the only other first-line treatments available are theophylline and aminophylline. Although theophylline has similar short-term efficacy to caffeine citrate, it has therapeutic disadvantages, including higher rates of toxicity than caffeine citrate (5, 6). In addition, neither theophylline nor aminophylline are approved for the management of apnea of prematurity in China. The use of theophylline or aminophylline as an active comparator would therefore be difficult to justify in this population—indeed, such a design could potentially also be argued as being unethical. The current single-arm design was therefore considered the most appropriate to address the overall study aim. Importantly, the study was specifically designed around the primary endpoint, which can be interpreted without a comparator group. Another aspect of the study that makes interpretation of the secondary endpoints challenging is the small proportion of neonates who required maintenance therapy for 2 or 4 weeks—this does however suggest that caffeine citrate provided a rapid improvement in apnea. Finally, although the study was multicentered, it was conducted in a single country. Although this limits the generalizability of the results, the rationale for conducting the study was driven by a post-approval regulatory request.

In conclusion, in this large, prospective, open-label study, premature infants with a history of apnea who received caffeine citrate were significantly less likely to experience future apnea events. The study helps to validate the recommendations to use caffeine citrate for such neonates.

## Data Availability Statement

The datasets generated for this study are available on submission of a valid research proposal to the corresponding author.

## Ethics Statement

The studies involving human participants were reviewed and approved by the independent ethics committees or research boards at each institution. Written informed consent to participate in this study was provided by the participants' parents or legal guardians.

## Author Contributions

AG, JL, DS, SN, and LF contributed to the conception and design of the study, and to the interpretation of the data. LD, XT, CC, and XG contributed to the acquisition and interpretation of the data. All authors revised the manuscript critically for intellectual content, provided final approval of the version to be published, and agree to be accountable for all aspects of the work.

### Conflict of Interest

LF, AG, JL, DS, and SN are employed by Chiesi, the sponsor of the study. LD, XT, CC, and XG declare that they have no competing financial interests that might have influenced the work described in this manuscript. The authors declare that this study received funding from Chiesi Farmaceutici SpA. Employees of the funder were involved in the conception and design of the study, to the interpretation of the data, and (as authors) revised the manuscript critically for intellectual content and provided final approval of the version to be published.
