# Supplementary material for: Caffeine Citrate for Apnea of Prematurity: A Prospective, Open-Label, Single-Arm Study in Chinese Neonates
Source: Front Pediatr. 2020 Mar 11;8:76. doi: 10.3389/fped.2020.00076 (PMC7078308; doi:10.3389/fped.2020.00076)
Supplement: Supplementary file 1 [file Data_Sheet_1.pdf]

Supplementary Material

1.1 Methods

1.1.1 Inclusion Criteria

Subjects eligible for enrolment in the study must meet all of the following criteria:

1. Written informed consent signed by infant's parents/legal representative (according to local regulation) prior to any study-related procedures.
2. Premature infants of either sex with a gestational age between 28 and 33 weeks, admitted to participating hospital.
3. First apnea event: breathing cessation for at least 20 seconds or less if accompanied by bradycardia (HR < 100 bpm) or oxygen desaturation (SpO2 < 80 %) occurring more than 12 h after birth.
4. At least three other apnea events within 24 h after the first occurrence.

1.1.2 Exclusion Criteria

Subjects meeting any of the following criteria must not be enrolled in the study:

Items from 2 to 9 applied to enrolment only (i.e., to be satisfied only within 5 days before Visit 2) for exclusion of secondary apnea:

1. Hematocrit (HCT) >65% or <40% in room air.
2. Clinical suspicion or proven sepsis.
3. Blood glucose <2.6 mmol/L (or 45 mg/dL).

4. Hypocalcemia (<1.0 mmol/L ionized calcium).
5. Infant considered for exchange transfusion for hyperbilirubinemia.
6. Blood urea nitrogen (BUN) >20 mg/dL or urine output <1 mL/kg/hour, the latter occurring at least 24 h after the birth.
7. Body temperature <36.0 or >38.5C.
8. Hemodynamically significant patent ductus arteriosus (PDA) confirmed by cardiac ultrasound (Ductus arteriosus internal diameter > 1.5mm, or left atrium to aorta diameter ratio ≥1.4, also that needs medication or surgical intervention).
9. Suspected or confirmed necrotizing enterocolitis by abdominal X ray.
10. Intraventricular hemorrhage (IVH) >grade 2 confirmed by cranial ultrasound.
11. Apnea resulting from congenital upper airway obstruction (e.g., Pierre-Robin syndrome).
12. Receiving assisted ventilation via an endotracheal tube or intermittent mandatory ventilation (IMV).
13. Subjects who have any other condition that in the investigators' opinion makes them unsuitable for participation in the study.

1.1.3 List of sites/ethics committees and investigators

- Children’s Hospital of Zhejiang University School of Medicine - Prof Lizhong Du
- Women’s Hospital of Zhejiang University School of Medicine - Mingyuan Wu
- Peking University Third Hospital - Xiaomei Tong
- Peking University First Hospital - Qi Feng
- Beijing Children's Hospital, Capital Medical University - Hong Liu
- West China Second Hospital, Sichuan University - Dapeng Chen
- Qilu Hospital of Shandong University - Wen Li
- Children's Hospital of Shanxi Province - Jianhong Yao
- Wuxi City People's Hospital - Min Yu
- First Hospital of Jilin University - Chaoying Yan
- Fudan University Children's Hospital - Chao Chen
- Shanghai Children's Hospital - Gang Qiu
- Shanghai Jiaotong University Affiliated Shanghai Children's Medical Center - Jianhua Sun
- Shanghai Jiaotong University School of Medicine Affiliated Xinhua Hospital - Jianxing Zhu
- Second Affiliated Hospital of Wenzhou Medical College - Zhenlang Lin
- Hunan Provincial People's Hospital - Aimin Zhang
- Hunan Children's Hospital - Xirong Gao
- Capital Medical University, Beijing Maternity Hospital - Jianrong Ma
- Binzhou Medical College Hospital - Xiuxiang Liu

1.2 Safety results

Nine neonates died during the study due to 12 adverse events, none considered related to caffeine citrate therapy. The adverse events leading to death were neonatal necrotizing enterocolitis (four events), neonatal sepsis (two events), peritonitis (one event), neonatal respiratory distress syndrome (one event), neonatal respiratory tract hemorrhage (one event), disseminated intravascular coagulation (one event), cardiac tamponade (one event) and sclerema (one event).
